# Protein structure and phenotypic analysis of pathogenic and population missense variants in *STXBP1*

**DOI:** 10.1002/mgg3.304

**Published:** 2017-06-20

**Authors:** Mohnish Suri, Jochem M. G. Evers, Roman A. Laskowski, Sinead O'Brien, Kate Baker, Jill Clayton‐Smith, Tabib Dabir, Dragana Josifova, Shelagh Joss, Bronwyn Kerr, Alison Kraus, Meriel McEntagart, Jenny Morton, Audrey Smith, Miranda Splitt, Janet M. Thornton, the DDD Study, Caroline F. Wright

**Affiliations:** ^1^ Nottingham Regional Genetics Service Nottingham University Hospitals NHS Trust City Hospital Campus, The Gables, Hucknall Road Nottingham NG5 1PB UK; ^2^ European Bioinformatics Institute (EMBL‐EBI) Wellcome Genome Campus, Hinxton Cambridge CB10 1SD UK; ^3^ MRC Cognition and Brain Sciences Unit 15 Chaucer Road Cambridge CB2 7EF UK; ^4^ Department of Medical Genetics University of Cambridge Cambridge Biomedical Campus Cambridge CB2 0QQ UK; ^5^ Manchester Centre for Genomic Medicine St Mary's Hospital, Central Manchester University Hospitals NHS Foundation Trust Manchester Academic Health Science Centre Manchester M13 9WL UK; ^6^ Northern Ireland Regional Genetics Centre Belfast Health and Social Care Trust Belfast City Hospital Lisburn Road Belfast BT9 7AB UK; ^7^ South East Thames Regional Genetics Centre Guy's and St Thomas’ NHS Foundation Trust Guy's Hospital Great Maze Pond London SE1 9RT UK; ^8^ West of Scotland Genetics Service Queen Elizabeth University Hospital Laboratory Medicine Building Glasgow G51 4TF UK; ^9^ Yorkshire Regional Genetics Service Department of Clinical Genetics Leeds Teaching Hospitals NHS Trust Chapel Allerton Hospital Chapeltown Road Leeds LS7 4SA UK; ^10^ South West Thames Regional Genetics Centre St George's Healthcare NHS Trust St George's University of London Cranmer Terrace London SW17 0RE UK; ^11^ West Midlands Regional Clinical Genetics Service and Birmingham Health Partners Birmingham Women's and Children's NHS Foundation Trust Birmingham Women's Hospital Mindelsohn Way, Edgbaston Birmingham B15 2TG UK; ^12^ Northern Genetics Service Newcastle upon Tyne Hospitals NHS Foundation Trust Institute of Human Genetics International Centre for Life Central Parkway Newcastle upon Tyne NE1 3BZ UK; ^13^ Wellcome Trust Sanger Institute Wellcome Genome Campus, Hinxton Cambridge CB1 8RQ UK; ^14^ University of Exeter Medical School Royal Devon & Exeter Hospital Barrack Road Exeter EX2 5DW UK

**Keywords:** Epilepsy, Exome Aggregation Consortium, genomics, Munc18, protein structure, syntaxin‐binding protein 1

## Abstract

**Background:**

Syntaxin‐binding protein 1, encoded by *STXBP1*, is highly expressed in the brain and involved in fusing synaptic vesicles with the plasma membrane. Studies have shown that pathogenic loss‐of‐function variants in this gene result in various types of epilepsies, mostly beginning early in life. We were interested to model pathogenic missense variants on the protein structure to investigate the mechanism of pathogenicity and genotype–phenotype correlations.

**Methods:**

We report 11 patients with pathogenic de novo mutations in *STXBP1* identified in the first 4293 trios of the Deciphering Developmental Disorder (DDD) study, including six missense variants. We analyzed the structural locations of the pathogenic missense variants from this study and the literature, as well as population missense variants extracted from Exome Aggregation Consortium (ExAC).

**Results:**

Pathogenic variants are significantly more likely to occur at highly conserved locations than population variants, and be buried inside the protein domain. Pathogenic mutations are also more likely to destabilize the domain structure compared with population variants, increasing the proportion of (partially) unfolded domains that are prone to aggregation or degradation. We were unable to detect any genotype–phenotype correlation, but unlike previously reported cases, most of the DDD patients with *STXBP1* pathogenic variants did not present with very early‐onset or severe epilepsy and encephalopathy, though all have developmental delay with intellectual disability and most display behavioral problems and suffered seizures in later childhood.

**Conclusion:**

Variants across *STXBP1* that cause loss of function can result in severe intellectual disability with or without seizures, consistent with a haploinsufficiency mechanism. Pathogenic missense mutations act through destabilization of the protein domain, making it prone to aggregation or degradation. The presence or absence of early seizures may reflect ascertainment bias in the literature as well as the broad recruitment strategy of the DDD study.

## Introduction

Brain function relies on the release of neurotransmitters from chemical synapses, which in turn relies on the rapid and regulated fusion of synaptic vesicles with the plasma membrane. The central machinery involved in the fusion process is composed of two conserved protein families: the SNARE (*s*oluble *N*‐ethylmaleimide‐sensitive factor *a*ttachment *re*ceptor) and the SM (Sec1/Munc18) proteins (Südhof and Rothman 2009), now known as syntaxin‐binding proteins. Heterozygous pathogenic variants in syntaxin‐binding protein 1 (STXBP1, previously called Munc18‐1), encoded by the *STXBP1* gene, have been reported in nearly 200 patients with various types of developmental disorders, primarily epilepsy/epileptic encephalopathy, developmental delay, and intellectual disability (Saitsu et al. 2008, 2010; Otsuka et al. 2010; Milh et al. 2011; Neale et al. 2012; Epi 4K Consortium and Epilepsy Phenome/Genome Project 2013; Romaniello et al. 2014, 2015; Weckhuysen et al. 2013; Barcia et al. 2014; Carvill et al. 2014; Michaud et al. 2014; Tso et al. 2014; Keogh et al. 2015; Khaikin and Mercimek‐Mahmutoglu 2016; Stamberger et al. 2016). Although the mechanism of disease is consistent with autosomal dominant haploinsufficiency, the mode by which pathogenic missense variants cause disease still remains unclear.

**Figure 1:**
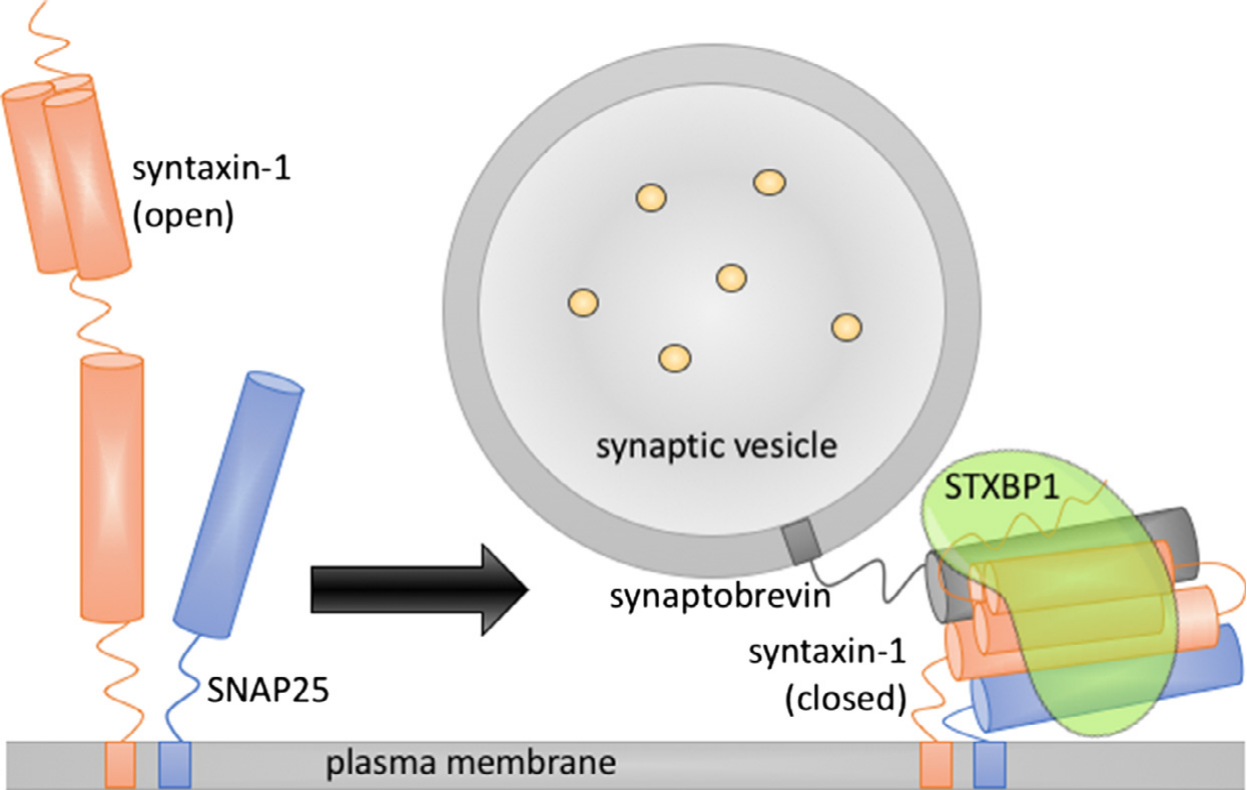
Cartoon representation of the role of STXBP1 in priming vesicle fusion, showing binding to syntaxin‐1 (closed conformation) and other members of the SNARE complex.

Many details about the molecular basis of membrane fusion remain to be established (Rizo and Xu 2015), but it is believed that STXBP1 plays an essential role in synaptic vesicle docking, priming, and fusion (Toonen and Verhage 2007; Südhof and Rothman 2009; Carr and Rizo 2010), and is highly expressed in the brain (Uhlén et al. 2015). To mediate these different functions, STXBP1 is involved in multiple types of interaction with SNAREs, of which the interaction with syntaxin‐1 is the most important. Syntaxin‐1 can be folded in an open and closed conformation. In the closed conformation, a short sequence at the N‐terminus (the N‐peptide) and an N‐terminal three helix bundle (the H_*abc*_ domain) are held in a compact autoinhibitory conformation that hinders interactions with other SNAREs to form the SNARE complex. STXBP1 initially binds syntaxin‐1 in the closed conformation, accounting for the regulating function of the protein. Studies have suggested it is responsible for transporting syntaxin‐1 to the plasma membrane in the closed conformation, where it facilitates vesicle fusion (Fig. 1) (Arunachalam et al. 2008; Han et al. 2009; Ma et al. 2011; Martin et al. 2013).

**Figure 2:**
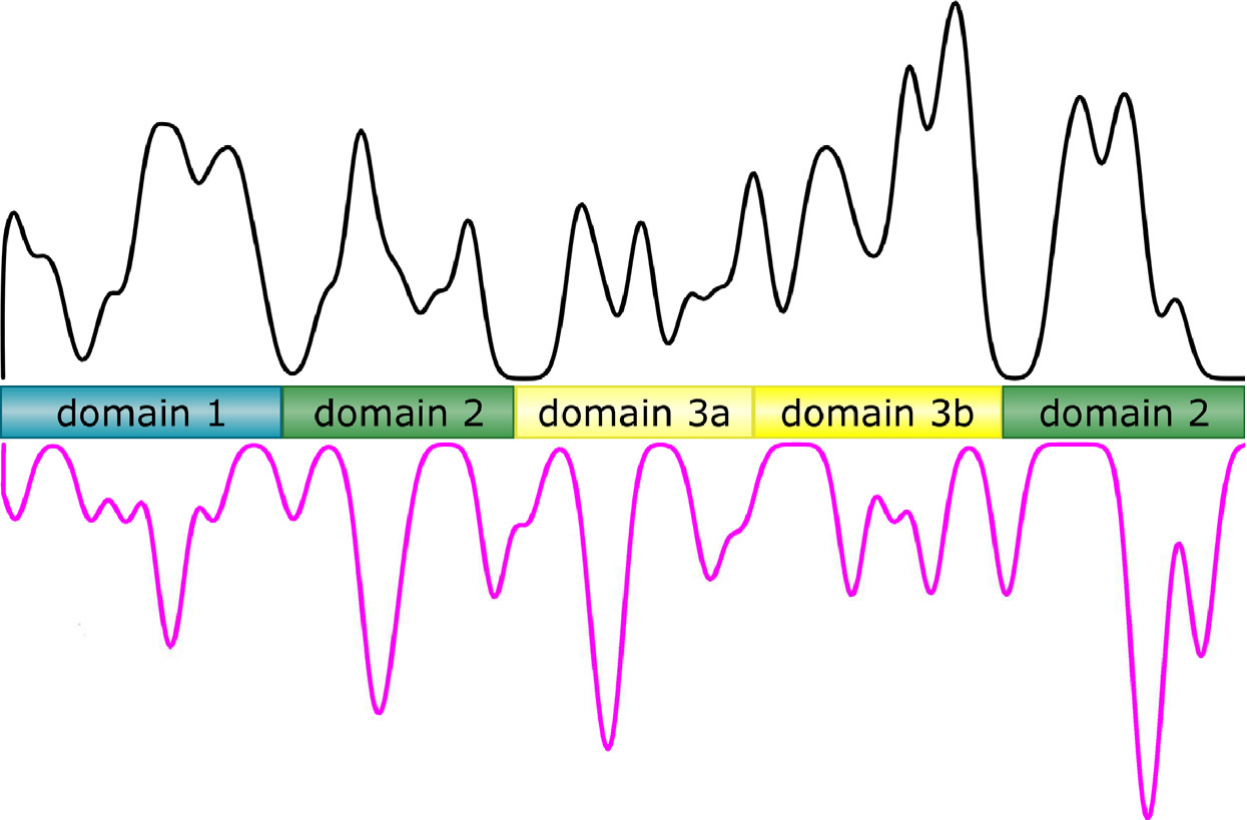
Domain architecture of syntaxin‐binding protein 1. The distribution of the population missense variants from ExAC are shown as a density plot on top (black) and is based on 65 unique missense variants; the bottom distribution shows all pathogenic variants from this study and the literature (magenta), and is based on 40 unique missense variants.

The crystal structure of STXBP1 in complex with syntaxin‐1 shows that it binds in the closed conformation to the syntaxin‐1 N‐peptide and the H_*abc*_ domain, PDB accession *3c98* (Misura et al. 2000; Burkhardt et al. 2008). STXBP1 adopts an arch‐shaped structure consisting of three domains: domain 1, domain 2, and domain 3, where domain 3 is subdivided into domain 3a and 3b, assigned by Misura et al. (2000) (Fig. 2). Domain 1 and domain 3a form the cavity that binds the H_*abc*_ domain and the SNARE motif of syntaxin‐1. Furthermore, the opposite side of domain 1 binds to the N‐peptide. STXBP1 binds to the four helix bundle of the SNARE complex (Dulubova et al. 2007; Shen et al. 2007) and there is evidence that this binding is mediated by the same cavity that binds syntaxin‐1 (Shen et al. 2010; Xu et al. 2010) as well as domain 3a (Hu et al. 2011; Parisotto et al. 2014). Experiments with the STXBP1 homolog in yeast (Sec1p) suggested that the binding takes place via the groove between domains 1 and 2 (Hashizume et al. 2009; Weber‐Boyvat et al. 2016). Every domain of STXBP1 therefore plays a putative role in its function.

This study aims to further explain why pathogenic variants in STXBP1 lead to disease and hopefully thereby gain a better understanding of the protein. Here we describe 11 new patients from the Deciphering Developmental Disorder (DDD) study with pathogenic de novo mutations in *STXBP1* (Deciphering Developmental Disorders Study 2017). We provide detailed clinical data and analyze the structural consequences of the de novo missense mutations. We will also compare pathogenic missense variants from the literature with the population missense variants from the Exome Aggregation Consortium (ExAC) (Lek et al. 2016), which is depleted of severe pediatric conditions and therefore assumed not to have individuals with severe intellectual disability (ID) or significant developmental delay, to investigate the differences between tolerated and nontolerated missense variants from a protein structure perspective.

## Materials and Methods

### Ethical compliance

The DDD study has UK Research Ethics Committee approval (10/H0305/83, granted by the Cambridge South REC, and GEN/284/12 granted by the Republic of Ireland REC). Postdiagnostic phenotyping was carried out within the Phenotypes in Intellectual Disability study, with independent Ethics Committee approval (11/EE/0330, granted by Cambridge Central REC).

### Clinical and genomic data

All patients were recruited as family trios into the DDD study from Regional Genetic Services across the United Kingdom because of an unexplained developmental disorder, including severe neurodevelopmental disorders, and/or congenital anomalies, abnormal growth parameters, dysmorphic features, and unusual behavioral phenotypes. Genome‐wide microarray and whole exome sequencing were performed on all patients as detailed previously (Deciphering Developmental Disorders Study 2015). De novo mutations in *STXBP1* (NM_003165.3; ENSG00000136854) were identified in 11 of the 4293 probands (0.26%) using trio exome sequencing, and deposited into the DECIPHER database (Bragin et al. 2014). The results were subsequently validated in diagnostic molecular genetic laboratories by Sanger sequencing, and given to the families via their clinical teams.

Clinical characteristics at the time of recruitment to the DDD project were provided by referring clinical geneticists via the DECIPHER website (Bragin et al. 2014) using HPO terminology (Koehler et al. 2014). Diagnosed patients were subsequently invited to participate in detailed research phenotyping, and 10 patients participated. The assessment protocol included a structured medical history interview, neurological examination, assessment of global and specific functional abilities (Vineland Adaptive Behavior Scales), and standardized behavior questionnaire appropriate for young people with ID (Developmental Behavior Checklist) (Einfeld and Tonge 2002).

### Structural analysis

The structural analysis was performed on PDB accession *3c98* (Misura et al. 2000; Burkhardt et al. 2008), which contains the rat structure of STXBP1 (100% sequence identity to the human protein) in complex with the N‐terminal part of syntaxin‐1. The structures were analyzed and figures made using CCP4mg (McNicholas et al. 2011). The structural analysis of *STXBP2* (64% sequence identity to *STXBP1*) was performed on PDB accession *4cca*. The structures were superposed using the *superpose* function from the CCP4 suite to compare residue coordinates. The *superpose* function is based on secondary structure and iterative three‐dimensional alignment of protein backbone C‐alpha atoms (Krissinel and Henrick 2004). Residue conservation was retrieved from ConSurf (Dodge et al. 1998; Ashkenazy et al. 2010), and used 150 sequences predicted to be structurally similar to PDB accession *3c98*. Residue accessibility, a measure of how buried individual amino acids are within the domain structure, was calculated using Naccess (Hubbard and Thornton 1993). The change in thermodynamic stability upon mutation and the number of amino acid interactions were calculated using FoldX PositionScan and PrintNetworks commands (Schymkowitz et al. 2005).

Population missense variants, 71 in total, were retrieved from the ExAC, MA (http://exac.broadinstitute.org). Read data were manually checked for all variants to exclude mosaic variants. The ExAC lists pathogenic variants in the second isoform of *STXBP1* (Uniprot identifier: P61764‐2 instead of P61764‐1), where the last 18 amino acids are replaced by 27 different ones. Since the structure is based on the first isoform, the six variants in the last part of the protein were discarded. This leaves 65 population variants to be used for analysis.

## Results

### Patient data

Table 1 lists detailed phenotypes of all 11 patients with de novo *STXBP1* mutations identified by the DDD study, with DECIPHER IDs (Bragin et al. 2014). Seven patients were female and four were male; their age range was 1.6–15.5 years. Only two were born prematurely (<37 weeks completed gestational age). None of the patients had intrauterine growth retardation and only one patient had microcephaly (head circumference <2nd centile). Although six patients had some facial dysmorphism, there were no consistent dysmorphic findings in this group. Other low‐frequency findings in these patients included gastroesophageal reflux, tapered fingers, eye problems, and joint laxity.

**Table 1:** Detailed clinical phenotypes of all 11 patients with de novo *STXBP1* pathogenic variants identified in the first 4293 trios from the DDD Study

DECIPHER ID	258815	261841	260459	261220	265950	270001	261234	273873	258242	263903	272650
Age at recruitment (age at last clinical assessment)	6.41 (13)	8.21 (12.75)	11.97	7.56	10.78	1.93	12.01 (15.5)	1.61	11.32	10.27 (15.1)	6.5 (12.92)
Sex	F	F	F	M	F	F	M	F	M	M	F
Gestation (weeks)	40	39	38	41	39	40	33	40	35	42	42
Birth weight (centile), kg	3.544 (62)	3.373 (65)	4.053 (99)	4.45 (96)	2.8 (17)	3.46 (55)	1.9 (34)	3.2 (33)	2.5 (54)	3.86 (73)	3.2 (33)
Last recorded OFC (centile), cm	51 (13)	54 (78)	56 (90)	54 (57)	48 (1)	46.5 (2)	55 (48)	47 (16)	56.2 (79)	54 (36)	52.5 (62)
Developmental delay	Yes	Yes	Yes	Yes	Yes	Yes	Yes	Yes	Yes	Yes	Yes
Age at independent walking	2.1 years	8 years	Unknown	Unknown	2.5–3 years	Unknown	3 years	Unknown	Unknown	2.5–3 years	Unknown
S&L development	First words 2–2.5 years. Can speak in short sentences	No speech	No speech	No speech	No speech	No speech	Single words from 2.5 years, limited vocabulary of single words only	First words 20 months	First words 8 months	First words 5 years	First words 3–5, can use words and short sentences
ID (VABC score)	Yes (47)	Yes (33)	Yes	Yes	Yes	Unknown	Yes (30)	Yes	Yes	Yes (31)	Yes (45)
Behavioral problems	Anxiety and phobia	Stereotypic behavior, sociable, mild anxiety	Unknown	Autism	Stereotypic behavior	Aggressive and impulsive behavior, bruxism	Aggressive and difficult behavior	Unknown	Echolalia	Autism hand‐flapping, intermittent hyperventilation, short attention span, sociable	Sensory hypersensitivity, separation anxiety, some aggressive behaviors
Developmental Behavioral Checklist total problem behavior centile (stratified by age and ID severity)	90	90					94			52	92
Seizures	Absence seizures, single tonic–clonic generalized seizure at age 10 years	No (unilateral “twitching” and clenched hands during infancy only)	Yes (complex partial)	No	1–2 per month < 1 min generalized tonic–clonic	No	Complex partial, frequent and brief from age 15, worsened on risperidone	Infantile encephalopathy (severe seizure disorder in the first year of life)	Focal seizures at 6 weeks, stopped after ~2 months	Infrequent generalized seizures (3 in total?), absence seizures	Possible febrile seizures at 8 months, complex partial seizures (clusters)
Facial dysmorphism	Down‐slanted palpebral fissures, anteverted nares, malar flattening	Round face, up‐slanted palpebral fissures, depressed nasal bridge, malar flattening, narrow mouth		Protruding ear		Frontal bossing, broad face, short nose, depressed nasal bridge, narrow mouth		Flat occiput	Deep philtrum, malar flattening, exaggerated cupid's bow		
Neurological findings	Fine tremor	Infantile axial hypotonia, tremor, broad‐based gait, poor coordination	Possible tremor	Tremor, truncal ataxia	Broad‐based gait, very poor coordination, intention tremor	Infantile hypotonia, pain insensitivity	None	Developmental regression, resting tremor, head titubation		Ataxia with broad‐based gait, unilateral hand tremor	Hypotonia, tremor
MRI brain	Normal	Normal	Normal	No	Abnormal bilateral symmetrical abnormal gray–white matter differentiation in anterior temporal lobes	Normal	Normal CT	Normal	Normal	Normal	Normal
*STXBP1* mutation	c.704G>A; p.Arg235Gln	c.1631G>T; p.Gly544Val	c.437_438delCCinsC; p.Leu147Trpfs*18	c.713_714delACinsA; p.Ser240Alafs*8	c.568C>T; p.Arg190Trp	c.568C>T; p.Arg190Trp	c.778G>T; p.Glu260*	c.1651C>T; p.Arg551Cys	c.148dupA; p.Ile50Asnfs*14	c.1099C>T; p.Arg367*	c.533C>T; p.Thr178Ile
Other features	Gastroesophageal reflux and sleep disturbances in infancy	Astigmatism, tapered fingers, inverted nipples, increased body weight	Tapered fingers	Joint laxity	Bilateral moderate ensorineural hearing impairment, divergent squint, menarche at 9 years	Gastroesophageal reflux, constipation, broad palm, tapered fingers, broad hallux, squint	Talipes, bilateral inguinal hernia, difficult weaning, placid baby	Constipation		Well, placid baby, high‐pitched voice	Joint laxity

F, female; M, male; OFC, orbitofrontal cortex circumference; ID, intellectual disability; VABC, Vineland Adaptive Behavior Scales.

All patients had developmental delay, with age range for starting to walk independently from 2.1 to 8 years. All patients had ID with speech and language delay, and four patients have not yet developed speech. Only one patient developed speech at an appropriate age, though it is still very limited. Six patients were assessed using the Vineland Adaptive Behavior Scales (parental interview) and all had low scores with four in the severe ID range (26, 33, 30, and 31) and two in the moderate ID range (47 and 45). On average, communication skills and daily living skills were as expected for global ability, whereas motor skills and social abilities were slightly stronger than expected. Eight patients had behavioral problems, three having some form of anxiety and three having stereotypies or repetitive behaviors such as hand flapping. Two patients had been diagnosed with an autism spectrum disorder and three patients showed some aggressive behaviors. Where available and with one exception, standardized questionnaire ratings indicated much higher risk of behavior problems than expected for age or ID severity, with highest scores in the self‐absorbed, disruptive, and communication disturbance domains.

Eight patients had a history of epilepsy. Seizure types, severity, and age of onset varied widely within the group. One patient had severe infantile encephalopathy, one patient had absence seizures with a single generalized tonic–clonic seizure, one patient had absence seizures with several generalized seizures, three patients had complex partial seizures, and two patients had seizures with no further details available. An additional patient also had unilateral twitching and clenched hands during infancy only, though it is unclear if these were seizures. The remaining two patients had not had any seizures at the last clinical assessment. Only one of the patients had early infantile epileptic encephalopathy. Nine patients had abnormal neurological findings, particularly tremor, which was seen in six patients. Hypotonia and ataxia were each seen in three patients. Ten patients had neuroimaging (MRI brain in nine and CT brain in one), but only one MRI brain scan was reported to be abnormal (bilateral symmetrical abnormalities in anterior temporal lobe).

### Variant data and comparative protein modeling

The 11 de novo *STXBP1* mutations identified in DDD include three frameshift, two nonsense, and six missense changes (Table 1; note that the same missense pathogenic variant was present in two unrelated patients). Table 2 lists the results of standard in silico analyses of the pathogenicity of the five missense variants identified in our patients. Four of these variants have been reported previously, all in patients with early‐onset epileptic encephalopathy. The c.533C>T; p.(Thr178Ile) variant has not been reported previously.

**Table 2:** Analysis of the five missense variants identified in *STXBP1* in the DDD Study

DECIPHER ID	258815	261841	265950 and 270001	273873	272650
*STXBP1* mutation	c.704G>A; p.Arg235Gln	c.1631G>T; p.Gly544Val	c.568C>T; p.Arg190Trp	c.1651C>T; p.Arg551Cys	c.533C>T; p.Thr178Ile
PhyloP	5.69	5.61	2.38	5.61	5.61
Grantham distance	43	109	101	180	89
Align GVGD	C35	C0	C65	C0	C15
SIFT	0	0	0	0	0
PROVEAN	Damaging	Damaging	Damaging	Damaging	Damaging
Polyphen 2	0.994	1	0.999	0.897	0.535
Mutation taster	Disease causing	Disease causing	Disease causing	Disease causing	Disease causing
SNPs&GO	Disease related	Disease related	Disease related	Disease related	Neutral
ClinVar					
dbSNP	c.704G>A; p.Arg235Gln (rs794727970)	Not present	c.568C>T; p.Arg190Trp (rs796053355 ‐ pathogenic)	Not present	Not present
ExAC	Not present	Not present	Not present	Not present	Not present
Protein domain	2	2	2	2	2
Literature (Phenotype)	Stamberger et al. (2016) (EOEE)	Weckhuysen et al. (2013) (EOEE→West)	Carvill et al. (2014)(EOEE); Epi 4k Consortium 2014 (EOEE→?LSG)	Neale et al. (2012) (autism study); Weckhuysen et al. (2013) (EOEE); Stamberger et al. (2016) (2 patients both with EOEE)	Not previously reported

PhyloP (Pollard et al. 2010), Grantham distance (Grantham 1974), Align GVGD (Tavtigian et al. 2006), SIFT (Kumar et al. 2009), PROVEAN (Choi et al. 2012), Polyphen‐2 (Adzhubei et al. 2010), Mutation Taster (Schwarz et al. 2010), SNPs&Go (Calabrese et al. 2009), dbSNP (Sherry et al. 2001), ExAC (Lek et al. 2016). EOEE, early‐onset epileptic encephalopathy; LGS, Lennox–Gastaut syndrome.

**Figure 3:**
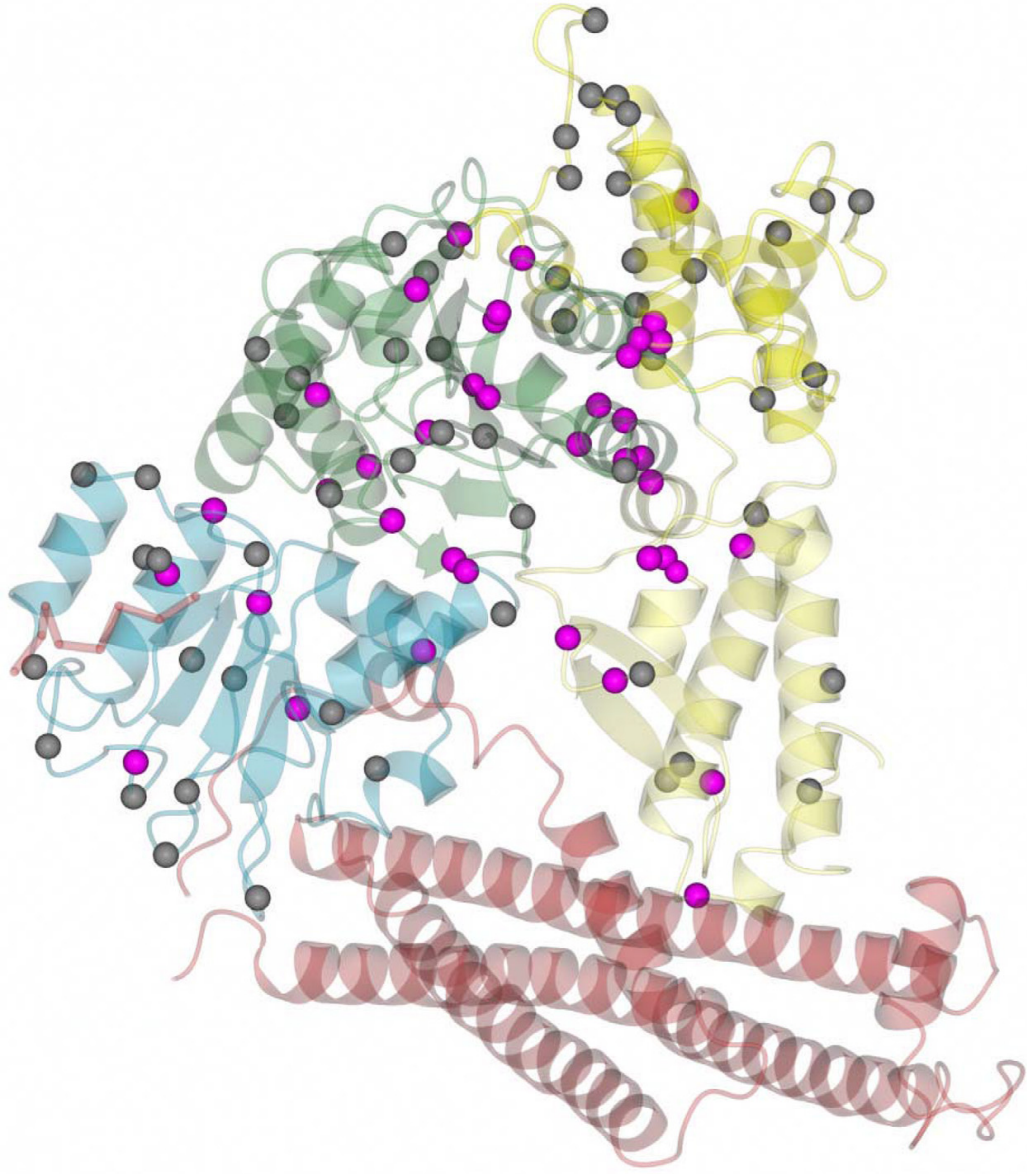
Ribbon diagram of syntaxin‐binding protein 1 (transparent, colored by domain as in Figure 1) in complex with part of syntaxin‐1 (red) with the C*α* positions of pathogenic missense variants (magenta) and population missense variants (gray). The N‐peptide is shown in the cylinder representation (bottom left).

The literature contains over 50 reported missense variants in *STXBP1* resulting in developmental disorders (summarized in Stamberger et al. 2016), and there are a further 65 population missense variants retrieved from the ExAC database (Lek et al. 2016), which are presumed to be benign with respect to severe childhood disorders. The location of unique missense variants in these two datasets – disease‐causing and population – is shown in the two‐dimensional (2D) protein domain structure in Figure 2 and the three‐dimensional (3D) protein structure in Figure 3. Both types of missense variants are scattered throughout the different structural domains of the protein, with no significant enrichment of disease‐causing variants in any specific area of the protein, reflecting the fact that *STXBP1* is a highly constrained gene, with fewer loss‐of‐function variants (pLI = 1) and missense variants (*z* = 5.22) than expected throughout the gene (Lek et al. 2016).

**Figure 4:**
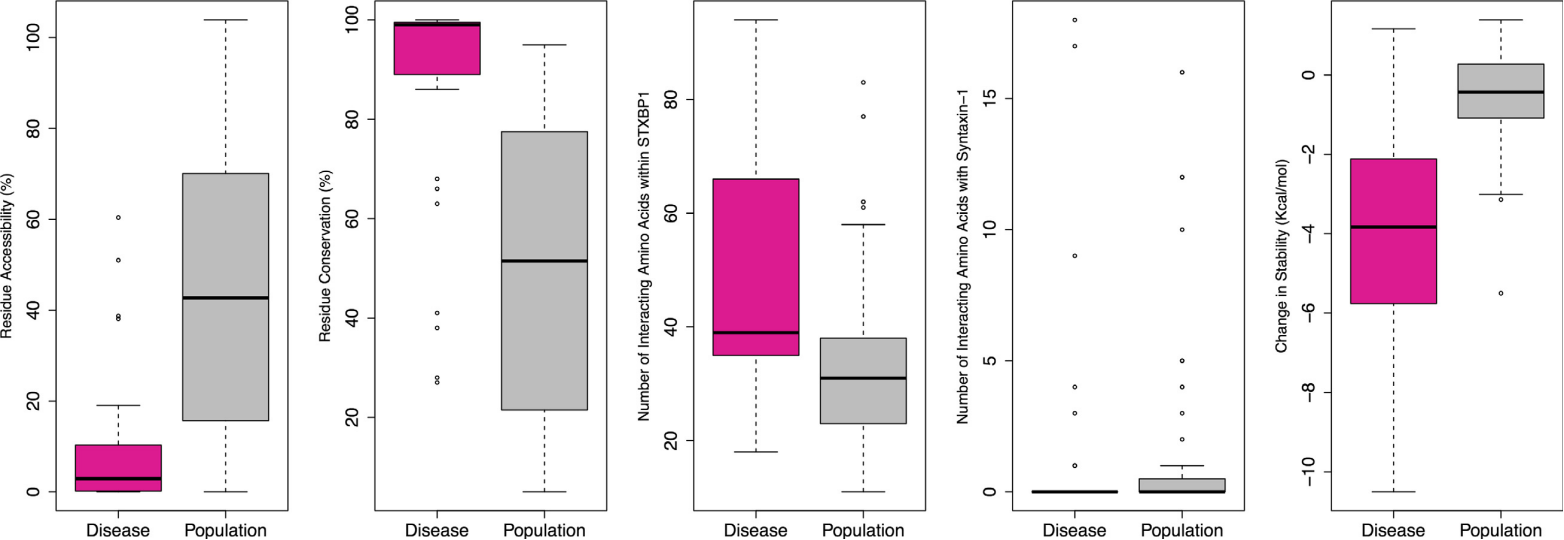
Boxplots of (A) residue accessiblity (Hubbard and Thornton 1993), (B) sequence conservation (Dodge et al. 1998; Ashkenazy et al. 2010), (C) number of interacting amino acids within the STXBP1 domain or (D) with syntaxin‐1, and (E) predicted change in stability of the protein domain (Schymkowitz et al. 2005) for pathogenic (pink) and population (gray) missense variants.

Amino acids that are altered in the population versus those that cause disease nonetheless differ significantly in terms of their residue accessibility (RSA) (Fig. 4A), their sequence conservation (Fig. 4B), the number of interacting amino acids within the STXBP1 domain (Fig. 4C), though not with syntaxin‐1 (Fig. 4D), and their predicted change in structural stability upon mutation (Fig. 4E). The RSA of the disease‐associated pathogenic variants is significantly lower than that of the population variants and of all residues in the protein (Wilcoxon–Mann–Whitney test, *P* = 6e‐8 and *P* = 2e‐5, respectively), while the population variants are also significantly different from all residues in the protein (*P* = 2e‐5). This indicates that the pathogenic changes are significantly more often located in the core of the protein, while the population variants are more likely to occur on the surface, as has been noted in the 1000 genomes project (de Beer et al. 2013). Additionally, the residue conservation of the disease‐causing variants is significantly higher than that of the population variants and of all residues in the protein (*P* = 1e‐9 and *P* = 8e‐7, respectively), while the conservation of the population variants is significantly lower than all residues in the protein (*P* = 3e‐6). The amino acids where pathogenic variants occur have significantly more interactions with other amino acids in STXBP1 than those where population variants occur (*P* = 5e‐5), though there is no difference in interactions with syntaxin‐1. Pathogenic variants are predicted to be significantly more destabilized, relative to the wild type, than population variants (*P* = 7e‐10).

Interestingly, variants of Val84 and His445 occur in both the patient and population datasets, though different amino acids are substituted between the datasets: Val84Ile and His445Tyr (number of alleles present in ExAC was 54 and 1, respectively; Grantham distances of 29 and 83, respectively) occurred in developmentally normal individuals, whereas Val84Asp and His445Pro (Grantham distances of 121 and 77, respectively) were found to cause developmental problems in children. At position 84, isoleucine (the population variant) is present in 30% of the 150 aligned sequences used for conservation analysis (Ashkenazy et al. 2010), while aspartic acid (the pathogenic variant) is not present in any of the aligned sequences; in addition, isoleucine shares many amino acid properties with valine, while aspartic acid is very different being both larger and negatively charged. At position 445, histidine is only present in 28% of aligned sequences and, although the location tolerates a wide variety of amino acids, tyrosine (the population variant) is present in 14% of alignments, while proline (the pathogenic variant) is never present. Moreover, since the residue is located in a helix, the restricted backbone φ and ψ angles necessitated by a proline residue would likely break or substantially destabilize the helix. In both cases (positions 84 and 445), the pathogenic variant is predicted to cause a substantial decrease in protein stability relative to the wild type, while the population variant is predicted to be slightly stabilizing. These examples demonstrate the value of protein structural analysis in predicting whether a specific pathogenic variant will be deleterious or not.

**Figure 5:**
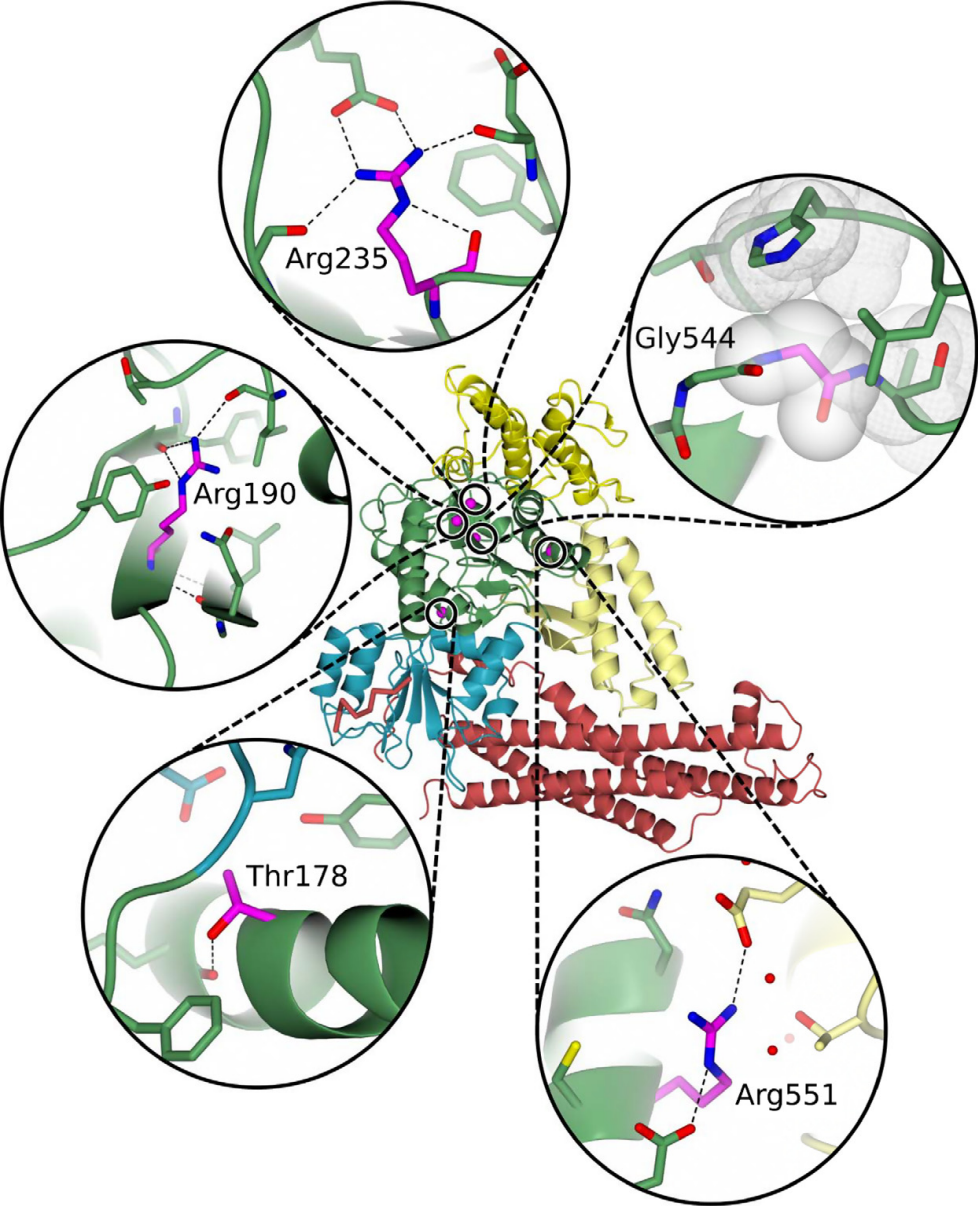
Structure of syntaxin‐binding protein 1 with the positions of the five missense pathogenic variants from the DDD study and their structural surroundings shown as insets.

The structural context of the five missense changes identified by the DDD study is shown in Figure 5, and their effect on the protein structure is considered in detail below. All the DDD variants are in domain 2 of the protein structure. Table 2 lists evidence for their pathogenicity.

- Thr178 (mutated to Ile) is positioned in the center of an α‐helix, where its side chain hydroxyl forms an hydrogen bond with the backbone oxygen of Gln175. This bond would be lost upon substitution to isoleucine, but it is uncertain if this causes the variant to be deleterious. Perhaps the slightly larger size of isoleucine destabilizes the protein fold. Thr178 is highly structurally conserved (99%) and the only other amino acid observed at this position is the even smaller amino acid alanine.
- Arg190 (mutated, in two unrelated DDD children, to Trp) is buried and forms three hydrogen bonds, two with the backbone oxygen of Tyr499 and one with the backbone oxygen of Leu494. This residue is 100% conserved in the alignment. Replacement by tryptophan will not only result in the loss of these hydrogen bonds, it will also disrupt the protein fold. Tryptophan is less flexible (two side chain torsion angles compared to four in arginine) and substantially larger, causing steric clashes with other residues in the core of the structure.
- Arg235 (mutated to Gln) is located in a loop and partakes in a network of electrostatic interactions. One of those interactions is a hydrogen bond between its own backbone oxygen and Nε. This is the only interaction that would remain upon changing arginine to glutamine; all others would be lost. Furthermore, arginine is the only residue that is able to occupy this very specific space, hence its 100% residue conservation.
- Gly544 (mutated to Val) resides in a loop and is preceded by Gly543. Both the residues are 100% structurally conserved and have φ and ψ angles (103° and 23° for Gly543 and 99° and 150° for Gly544) that other residues are unable to have. These torsion angles allow the loop to perfectly bridge the preceding β‐strand with the following α‐helix in this limited space. Moreover, substitution of Gly544 to valine would also result in a steric interference with the overlying loop. Substitution of this residue to aspartic acid and serine (Gly441Ser in *STXBP2*) has already been found to be deleterious for the protein (Cetica et al. 2010) indicating that any pathogenic variant of Gly544 is likely to result in loss of function.
- Arg551 (mutated to Cys) is sandwiched between two negatively charged residues, forming a salt bridge with one and a hydrogen bond with the other. Replacement to cysteine would result in a loss of these interactions and thereby compromise the stability of the protein. Arg551 is 99% conserved, indicating the importance of this residue at this position.

## Discussion

It is well established that heterozygous loss‐of‐function variants in *STXBP1* – including whole gene deletion, intragenic deletion, stop gain, frameshift, and splice donor/acceptor variants – result in a severe childhood developmental phenotype associated with seizures and ID. The gene is highly evolutionarily constrained, with just four apparent loss‐of‐function variants in the ExAC database of population variation (Lek et al. 2016). These four variants (three frameshift and one splice donor variant) are all located at very C‐terminal end of the protein, after the end of the structured domain, and absent from the first isoform, where they are unlikely to cause loss of function.

In order to try and elucidate the mechanism of pathogenicity of missense variants in *STXBP1*, we compared population missense variants (from ExAC) with pathogenic missense changes (from DDD and the literature). Although in principle the existence of a single population variant does not rule out pathogenicity, it is unlikely that the observed population variants in *STXBP1* are pathogenic, since severe early‐onset childhood disorders have specifically been excluded from ExAC. We evaluated missense changes in the 3D domain structure, but were unable to find any positional correlation with pathogenicity. Nonetheless, pathogenic missense variants are significantly more likely to be buried within the domain, at highly conserved residues that are involved in a network of intramolecular interactions within STXBP1 (Fig. 4). There is no evidence that pathogenic variants directly affect the binding interaction with syntaxin‐1. We also assessed the effect of both pathogenic and population variants on the predicted thermodynamic stability of the protein domain, relative to the wild type (Schymkowitz et al. 2005), and found that the pathogenic changes were significantly more likely to destabilize the domain (Fig. 4). This provides good evidence that the mechanism of pathogenicity for missense variants in *STXBP1* is haploinsufficiency, through destabilization of the native folded state of the protein domain, making it prone to misfolding, aggregation, and degradation (Nielsen et al. 2017). This mechanism has previously been suggested by Saitsu et al. (2012), where direct evidence for destabilization and aggregation was observed for several pathogenic missense variants in *STXBP1*.

Heterozygous pathogenic variants in the *STXBP1* gene can be associated with ID phenotypes, with or without epilepsy. The ID–epilepsy phenotypes include early infantile epileptic encephalopathy (EIEE) or Ohtahara syndrome (Saitsu et al. 2008), West syndrome (Deprez et al. 2010; Otsuka et al. 2010), Dravet syndrome (Carvill et al. 2014), infantile spasms (Mignot et al. 2011; Carvill et al. 2014; Michaud et al. 2014; Boutry‐Kryza et al. 2015), neonatal‐onset or early‐onset focal epilepsy (Vatta et al. 2012; Romaniello et al. 2014), partial complex epilepsy (Hamdan et al. 2011), and nonsyndromic epilepsy (Hamdan et al. 2009; Deprez et al. 2010). These phenotypes are invariably associated with ID and often with ataxia, tremor, and sometimes with a movement disorder (Kanazawa et al. 2010). Respiratory complex I and IV deficiency and lactic acidemia without respiratory complex deficiency have also been reported in single patients with *STXBP1*‐related epilepsy phenotypes (Barcia et al. 2014; Keogh et al. 2015; Li et al. 2016), as has atypical Rett syndrome (Olson et al. 2015; Romaniello et al. 2015).

However, intellectual disability phenotypes without seizures have only been reported previously in a small number of patients with heterozygous *STXBP1* pathogenic variants (Hamdan et al. 2011; Campbell et al. 2012; Rauch et al. 2012; Gburek‐Augustat et al. 2016; Stamberger et al. 2016). These patients usually have severe ID, often in combination with ataxia and tremor, with additional findings of autism, attention‐deficit disorder, and movement disorder in one or more patients. Stamberger et al. recently reviewed the phenotypic spectrum of 147 patients with *STXBP1* encephalopathy, including 45 previously unreported patients. A majority of these patients presented with early‐onset epilepsy and encephalopathy (EOEE) or Ohtahara syndrome. Only 4 of the 45 (<10%) previously unreported patients with *STXBP1* pathogenic variants had ID without seizures. Table S1 summarizes the clinical findings and *STXBP1* pathogenic variants in 12 previously reported patients with *STXBP1* pathogenic variants and ID without any seizures, as well as the two patients from our cohort.

Only 1 of the 11 probands reported here presented with an infantile epileptic encephalopathy phenotype, though another seven had some form of seizures and all had severe ID. Nonetheless, there is a clear underrepresentation of severe, early‐onset epilepsy phenotypes in our patient cohort relative to those previously published with mutations in *STXBP1*. This is unlikely to be related to the type of pathogenic variant or its location, as patients in our cohort had a range of stop gain, frameshift, and missense pathogenic variants spread across the protein. In addition, four of the five missense pathogenic variants that were identified in our cohort have been previously reported to cause early‐onset epileptic encephalopathy (Table 2). This may reflect variability of the phenotype associated with *STXBP1* pathogenic variants, or the modifying effects of variants in other genes. Interestingly, *STXBP1* knockout mice display a complete loss of neurotransmitter secretion from synaptic vesicles throughout development leading to neurodegeneration, though seizures have never been described (Verhage et al. 2000). Another possible explanation for the lack of a severe early‐onset epilepsy phenotype in our cohort could be ascertainment bias in the literature, which is absent from the DDD Study due to its wide eligibility criteria (Wright et al. 2015). We were unable to detect any genotype–phenotype correlation to explain the different presentation of these cases, and therefore suggest that *STXBP1* variants resulting in loss of function can cause a broad developmental phenotype associated with severe ID with or without a variety of other behavioral and neurological problems, particularly seizures/epilepsy, ataxia, and tremor.

In conclusion, analysis of de novo pathogenic variants in *STXBP1* in a cohort of children with developmental disorders confirms that loss‐of‐function variants in this gene cause severe developmental delay with or without seizures. Pathogenic variants in all domains of the protein encoded by *STXBP1* can result in the phenotype of ID with or without seizures, indicating that this phenotype does not correlate with the type or location of the pathogenic variant, but is consistent with a haploinsufficiency mechanism. Structural analysis of the STXBP1 confirms that the pathogenic missense variants are mostly buried inside the protein domain at highly conserved residues where a variant is likely to destabilize the domain and potentially lead to protein aggregation or degradation.

## Supporting information


**Table S1.** Summary of the clinical findings and *STXBP1* pathogenic variants in 12 previously reported patients with *STXBP1* pathogenic variants and ID without any seizures, as well as the two patients from the DDD cohort.
